# Dr. John J. Cullinane

**Published:** 1898-05

**Authors:** 


### OBITUARY.

---

Dr. John J. Cullinane, of Buffalo, died at his home, 180 Seneca street, March 31, 1898, aged 31 years. He came to this city from Watkins, N. Y., and graduated from the Buffalo University medical college in 1893. He was afterward appointed a member of the Fitch hospital staff. Subsequent to service in that capacity, he established himself in private practice and was one of the prominent young members of the profession. A promising future was suddenly terminated through his brief illness and death, the cause of which was attributed to acute pneumonia. His remains were taken to Watkins, N. Y., for interment, where his parents reside.

